# Consolidated Health Economic Evaluation Reporting Standards (CHEERS) statement

**DOI:** 10.1186/1478-7547-11-6

**Published:** 2013-03-25

**Authors:** Don Husereau, Michael Drummond, Stavros Petrou, Chris Carswell, David Moher, Dan Greenberg, Federico Augustovski, Andrew H Briggs, Josephine Mauskopf, Elizabeth Loder

**Affiliations:** 1 Institute of Health Economics, Edmonton, Canada; 2 Department of Epidemiology and Community Medicine, University of Ottawa, Ottawa, Canada; 3 University for Health Sciences, Medical Informatics and Technology, Hall in Tirol, Austria; 4 Centre for Health Economics, University of York, York, UK; 5 Warwick Medical School, University of Warwick, Coventry, UK; 6 Adis International, Auckland, New Zealand; 7 Clinical Epidemiology Program, Ottawa Hospital Research Institute, Ottawa, Canada; 8 Department of Health Systems Management, Faculty of Health Sciences, Ben-Gurion University of the Negev, Beer-Sheva, Israel; 9 Center for the Evaluation of Value and Risk in Health, Tufts Medical Center, Boston, MA, USA; 10 Health Economic Evaluation and Technology Assessment, Institute for Clinical Effectiveness and Health Policy (IECS), Buenos Aires, Argentina; 11 Universidad de Buenos Aires, Buenos Aires, Argentina; 12 Institute of Health and Wellbeing, University of Glasgow, Glasgow, Scotland, UK; 13 RTI Health Solutions, Research Triangle Park, NC, USA; 14 Division of Headache and Pain, Brigham and Women’s/Faulkner Neurology, Faulkner Hospital, Boston, MA, USA; 15 D Husereau, 879 Winnington Ave, Ottawa, ON K2B 5C4, Canada; 16 Clinical Epidemiology Editor, BMJ, London, UK

## Abstract

Economic evaluations of health interventions pose a particular challenge for reporting. There is also a need to consolidate and update existing guidelines and promote their use in a user friendly manner. The Consolidated Health Economic Evaluation Reporting Standards (CHEERS) statement is an attempt to consolidate and update previous health economic evaluation guidelines efforts into one current, useful reporting guidance. The primary audiences for the CHEERS statement are researchers reporting economic evaluations and the editors and peer reviewers assessing them for publication.

The need for new reporting guidance was identified by a survey of medical editors. A list of possible items based on a systematic review was created. A two round, modified Delphi panel consisting of representatives from academia, clinical practice, industry, government, and the editorial community was conducted. Out of 44 candidate items, 24 items and accompanying recommendations were developed. The recommendations are contained in a user friendly, 24 item checklist. A copy of the statement, accompanying checklist, and this report can be found on the ISPOR Health Economic Evaluations Publication Guidelines Task Force website (http://www.ispor.org/TaskForces/EconomicPubGuidelines.asp).

We hope CHEERS will lead to better reporting, and ultimately, better health decisions. To facilitate dissemination and uptake, the CHEERS statement is being co-published across 10 health economics and medical journals. We encourage other journals and groups, to endorse CHEERS. The author team plans to review the checklist for an update in five years.

## Background

**Figure 1:**
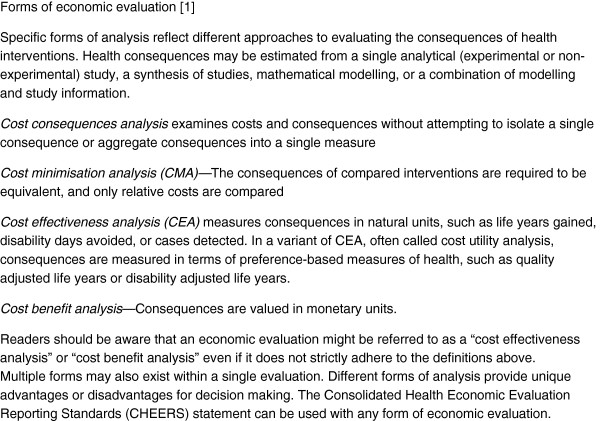
Forms of economic evaluation.

Health economic evaluations are conducted to inform resource allocation decisions. Economic evaluation has been defined as “the comparative analysis of alternative courses of action in terms of both their costs and their consequences” [1]. All economic evaluations assess costs, but approaches to measuring and valuing the consequences of health interventions may differ (see Figure 1).

Economic evaluations have been widely applied in health policy, including the assessment of prevention programmes (such as vaccination, screening, and health promotion), diagnostics, treatment interventions (such as drugs and surgical procedures), organisation of care, and rehabilitation. Economic evaluations are increasingly being used for decision making and are an important component of programmes for health technology assessment internationally [2].

### Reporting challenges and shortcomings in health economic evaluations

Compared with clinical studies, which report the consequences of an intervention only, economic evaluations require more reporting space for additional items, such as resource use, costs, preference related information, and cost effectiveness results. This creates challenges for editors, reviewers, and those who wish to scrutinise a study’s findings [3]. There is evidence that the quality of reporting of economic evaluations varies widely and could potentially benefit from improved quality assurance mechanisms [4,5].

With the increasing number of publications available, and opportunity costs from decisions based on misleading study findings, transparency and clarity in reporting are important. In addition, outside of economic evaluations conducted alongside clinical trials, there are no widespread mechanisms for warehousing economic evaluation data to allow for independent interrogation, such as ethics review proceedings, regulator dossiers, or study registries. Instead, independent analysis may rely on the record keeping of individual investigators.

Even if measures to promote transparency exist, such as registries, biomedical journal editors have increasingly promoted and endorsed the use of reporting guidelines. Endorsement of guidelines by journals for randomised controlled trials has been shown to improve reporting [6]. The combination of the risk of making costly decisions due to poor reporting with the lack of mechanisms that promote accountability makes transparency in reporting economic evaluations especially important and a primary concern among journal editors and decision makers [3,7].

### Aim and scope

The aim of the Consolidated Health Economic Evaluation Reporting Standards (CHEERS) statement is to provide recommendations, in the form of a checklist, to optimise reporting of health economic evaluations. The need for a contemporary reporting guidance for economic evaluations was recently identified by researchers and biomedical journal editors [8]. The CHEERS statement attempts to consolidate and update previous efforts [9-20] into a single useful reporting guidance.

The primary audiences for the CHEERS statement are researchers reporting economic evaluations and the editors and peer reviewers evaluating their publication potential. We hope the statement (which consists of a 24 item checklist and accompanying recommendations on the minimum amount of information to be included when reporting economic evaluations) is a useful and practical tool for these audiences and will improve reporting and, in turn, health and healthcare decisions. To best understand and apply the recommendations contained within the statement, we encourage readers to access the Explanation and Elaboration Report [21].

### Development of the CHEERS statement

The statement was developed by a task force supported by the International Society for Pharmacoeconomics and Outcomes Research (ISPOR), as part of a broader initiative to facilitate and encourage the interchange of expert knowledge and develop best practices. The CHEERS Task Force members were chosen by the chair of the task force primarily based on their longstanding academic expertise and contribution to the multidisciplinary field of health economic evaluation. In addition to four members of the task force with doctorates in economics and its sub-discipline of health economics (AHB, MD, JM, SP), members included experts in health technology assessment and decision making (FA, AHB, DH, MD, JM) and in clinical epidemiology and biostatistics (AHB, EL, DM), those in active clinical practice (EL, FA), and those with previous experience in reporting guideline development (MD, DM). All members are researchers in applied health and health policy, with five members currently serving as editors for journals in the field (AHB, CC, MD, DG, EL).

The CHEERS Task Force followed current recommendations for developing reporting guidelines [22]. Briefly, the need for new guidance was first identified through a survey of members of the World Association of Medical Editors. Of the 6% (55/965) who responded, 91% (n = 50) indicated they would use a standard if one were widely available [8]. Next, published checklists or guidance documents related to reporting economic evaluations were identified from a systematic review and survey of task force members [23]. Both of these activities were used to create a preliminary list of items to include when reporting economic evaluations. Recommendations of the minimum set of reporting items were then developed through a modified Delphi panel process. Forty eight individuals identified by the task force with broad geographical representation and representing academia, biomedical journal editors, the pharmaceutical industry, government decision makers, and those in clinical practice were invited to participate. Thirty seven agreed to participate. Participants were asked to score importance on a Likert scale and the average scores, weighted by each individual’s confidence in ability to score, were then used to rank items. A cut-off point was applied to the ranked list to determine the minimum number of items important for reporting.

The CHEERS statement recommendations have been independently reviewed and subsequently revised by task force members. The recommendations are entirely those of the task force—the sponsors of the study had no role in study design, data analysis, data interpretation, or writing of the final recommendations. A more complete description of the methods and findings of the Delphi panel are found in the larger explanation and elaboration document [21].

### Checklist items

The final recommendations are subdivided into six main categories: (1) title and abstract; (2) introduction; (3) methods; (4) results; (5) discussion; and (6) other. The recommendations are contained in a user friendly, 24 item checklist (Table 1) to aid users who wish to follow them. A copy of the checklist can also be found on the CHEERS Task Force website. (http://www.ispor.org/TaskForces/EconomicPubGuidelines.asp). In order to encourage dissemination and use of a single international standard for reporting, the task force approached 14 journals identified as either the largest publishers of economic evaluations or widely read by the medical and research community. Thirteen journals responded, and 10 expressed their ability and interest in endorsing this guidance. The CHEERS statement is being simultaneously published in *BMC Medicine*, *BMJ*, *BJOG: An International Journal of Obstetrics and Gynaecology*, *Clinical Therapeutics*, *Cost Effectiveness and Resource Allocation*, *The European Journal of Health Economics*, *International Journal of Technology Assessment in Health Care*, *Journal of Medical Economics*, *Pharmacoeconomics*, and *Value in Health*. To facilitate wider dissemination and uptake of this reporting guidance, we encourage other journals and groups to consider endorsing CHEERS.

**Table 1:** CHEERS checklist—Items to include when reporting economic evaluations of health interventions

**Section/item**	**Item No**	**Recommendation**	**Reported on page No/ line No**
**Title and abstract**			
Title	1	Identify the study as an economic evaluation or use more specific terms such as “cost-effectiveness analysis”, and describe the interventions compared.	___________________
Abstract	2	Provide a structured summary of objectives, perspective, setting, methods (including study design and inputs), results (including base case and uncertainty analyses), and conclusions.	___________________
**Introduction**			
Background and objectives	3	Provide an explicit statement of the broader context for the study.	
		Present the study question and its relevance for health policy or practice decisions.	___________________
**Methods**			
Target population and subgroups	4	Describe characteristics of the base case population and subgroups analysed, including why they were chosen.	___________________
Setting and location	5	State relevant aspects of the system(s) in which the decision(s) need(s) to be made.	___________________
Study perspective	6	Describe the perspective of the study and relate this to the costs being evaluated.	___________________
Comparators	7	Describe the interventions or strategies being compared and state why they were chosen.	___________________
Time horizon	8	State the time horizon(s) over which costs and consequences are being evaluated and say why appropriate.	___________________
Discount rate	9	Report the choice of discount rate(s) used for costs and outcomes and say why appropriate.	___________________
Choice of health outcomes	10	Describe what outcomes were used as the measure(s) of benefit in the evaluation and their relevance for the type of analysis performed.	___________________
Measurement of effectiveness	11a	*Single study-based estimates:* Describe fully the design features of the single effectiveness study and why the single study was a sufficient source of clinical effectiveness data.	___________________
	11b	*Synthesis-based estimates:* Describe fully the methods used for identification of included studies and synthesis of clinical effectiveness data.	___________________
Measurement and valuation of preference based outcomes	12	If applicable, describe the population and methods used to elicit preferences for outcomes.	___________________
Estimating resources and costs	13a	*Single study-based economic evaluation:* Describe approaches used to estimate resource use associated with the alternative interventions. Describe primary or secondary research methods for valuing each resource item in terms of its unit cost. Describe any adjustments made to approximate to opportunity costs.	___________________
	13b	*Model-based economic evaluation:* Describe approaches and data sources used to estimate resource use associated with model health states. Describe primary or secondary research methods for valuing each resource item in terms of its unit cost. Describe any adjustments made to approximate to opportunity costs.	___________________
Currency, price date, and conversion	14	Report the dates of the estimated resource quantities and unit costs. Describe methods for adjusting estimated unit costs to the year of reported costs if necessary. Describe methods for converting costs into a common currency base and the exchange rate.	___________________
Choice of model	15	Describe and give reasons for the specific type of decision-analytical model used. Providing a figure to show model structure is strongly recommended.	___________________
Assumptions	16	Describe all structural or other assumptions underpinning the decision-analytical model.	___________________
Analytical methods	17	Describe all analytical methods supporting the evaluation. This could include methods for dealing with skewed, missing, or censored data; extrapolation methods; methods for pooling data; approaches to validate or make adjustments (such as half cycle corrections) to a model; and methods for handling population heterogeneity and uncertainty.	___________________
**Results**			
Study parameters	18	Report the values, ranges, references, and, if used, probability distributions for all parameters. Report reasons or sources for distributions used to represent uncertainty where appropriate. Providing a table to show the input values is strongly recommended.	___________________
Incremental costs and outcomes	19	For each intervention, report mean values for the main categories of estimated costs and outcomes of interest, as well as mean differences between the comparator groups. If applicable, report incremental cost-effectiveness ratios.	___________________
Characterising uncertainty	20a	*Single study-based economic evaluation:* Describe the effects of sampling uncertainty for the estimated incremental cost and incremental effectiveness parameters, together with the impact of methodological assumptions (such as discount rate, study perspective).	___________________
	20b	*Model-based economic evaluation:* Describe the effects on the results of uncertainty for all input parameters, and uncertainty related to the structure of the model and assumptions.	___________________
Characterising heterogeneity	21	If applicable, report differences in costs, outcomes, or cost-effectiveness that can be explained by variations between subgroups of patients with different baseline characteristics or other observed variability in effects that are not reducible by more information.	___________________
**Discussion**			
Study findings, limitations, generalisability, and current knowledge	22	Summarise key study findings and describe how they support the conclusions reached. Discuss limitations and the generalisability of the findings and how the findings fit with current knowledge.	___________________
**Other**			
Source of funding	23	Describe how the study was funded and the role of the funder in the identification, design, conduct, and reporting of the analysis. Describe other non-monetary sources of support.	___________________
Conflicts of interest	24	Describe any potential for conflict of interest of study contributors in accordance with journal policy. In the absence of a journal policy, we recommend authors comply with International Committee of Medical Journal Editors recommendations.	___________________

For consistency, the CHEERS statement checklist format is based on the format of the CONSORT statement checklist.

### Concluding remarks

As the number of published health economic evaluations continues to grow, we believe more transparent and complete reporting of methods and findings will be increasingly important to facilitate interpretation and comparison of studies. We hope the CHEERS statement, consisting of recommendations in a 24 item checklist, will be viewed as an effective consolidation and update of previous efforts and serve as a starting point for standard reporting going forward.

We believe the CHEERS statement represents a considerable expansion over previous efforts. The strength of our approach is that it was developed in accordance with current recommendations for the development of reporting guidelines, using an international and multidisciplinary team of editors and content experts in economic evaluation and reporting [22]. Similar to the approach taken with other widely accepted guidelines, we have defined a minimum set of criteria though a modified Delphi technique and have translated these into recommendations, an explanatory document with explanations, and a checklist. Unlike some previous reporting guidance for economic evaluation, we have also made every effort to be neutral about the conduct of economic evaluation, allowing analysts the freedom to choose different methods.

There may be several limitations to our approach. A larger Delphi panel with a different composition could have led to a different final set of recommendations [24]. Some less common approaches and contexts (such as public health, developing countries, and system dynamic models) for conducting health economic evaluation may not be well represented by our sample of experts. Additionally, like many Delphi panel processes, we based decisions to reject or accept criteria on arbitrary levels of importance. However, we feel the group recruited to create the statement is sufficiently knowledgeable of the more common applications of economic evaluation, and the rules used to select criteria were created a priori and are consistent with previous efforts.

We believe it will be important to evaluate the effects of implementation of this statement and checklist on reporting in future economic evaluations. As methods for the conduct of economic evaluation continue to evolve, it will also be important to revisit or extend the guidance. The CHEERS Task Force feels that this statement should be reviewed for updating five years from its release.

## Competing interests

All authors have completed the ICMJE uniform disclosure form at http://www.icmje.org/coi_disclosure.pdf and declare: FA served as board member for the study funder; FA, AHB, CC, MD, DG, DH, EL, JM, and SP were provided support for travel to a face-to-face meeting to discuss the contents of the report; FA and MD have received payment from the study sponsor for serving as co-editors for *Value in Health*; no other relationships or activities that could appear to have influenced the submitted work. Elizabeth Loder is *BMJ* clinical epidemiology editor. She played no part in the peer review or decision making of this paper at the editorial level, and contributed solely as an author.

## Authors’ contributions

All authors provided a substantial contribution to the design and interpretation of the protocol and guidance, as well as writing sections of drafts, revising based on comments received, and approving the final version. DH conducted the analysis of Delphi panel responses, drafted and revised the protocol and the drafts of this paper, and is the guarantor for the study. All authors read and approved the final manuscript.

## References

[B1] Drummond MF Sculpher MJ Torrance G O’Brien J Stoddart GL Methods for the economic evaluation of health care programmes 2005 3 New York: Oxford University Press

[B2] Drummond MF Schwartz JS Jönsson B Luce BR Neumann PJ Siebert U Key principles for the improved conduct of health technology assessments for resource allocation decisions Int J Technol Assess Health Care 2008 24 244 258 18601792 10.1017/S0266462308080343

[B3] Rennie D Luft HS Pharmacoeconomic analyses JAMA 2000 283 2158 2160 10.1001/jama.283.16.2158 10791510

[B4] Neumann PJ Stone PW Chapman RH Sandberg EA Bell CM The quality of reporting in published cost-utility analyses, 1976–1997 Ann Intern Med 2000 132 964 10858180 10.7326/0003-4819-132-12-200006200-00007

[B5] Rosen AB Greenberg D Stone PW Olchanski NV Neumann PJ Quality of abstracts of papers reporting original cost-effectiveness analyses Med Decis Making 2005 25 424 428 10.1177/0272989X05278932 16061894

[B6] Turner L Shamseer L Altman DG Schulz KF Moher D Does use of the CONSORT Statement impact the completeness of reporting of randomised controlled trials published in medical journals? A Cochrane review. Syst Rev 2012 1 60 10.1186/2046-4053-1-60 23194585 PMC3564748

[B7] Drummond MF A reappraisal of economic evaluation of pharmaceuticals. Science or marketing? Pharmacoeconomics 1998 14 1 9 10182189 10.2165/00019053-199814010-00001

[B8] McGhan WF Al M Doshi JA Kamae I Marx SE Rindress D The ISPOR Good Practices for Quality Improvement of Cost-Effectiveness Research Task Force report Value Health 2009 12 1086 1099 10.1111/j.1524-4733.2009.00605.x 19744291

[B9] Task Force on Principles for Economic Analysis of Health Care Technology Economic analysis of health care technology. A report on principles Ann Intern Med 1995 123 61 70 7762918 10.7326/0003-4819-123-1-199507010-00011

[B10] Drummond MF Jefferson TO Guidelines for authors and peer reviewers of economic submissions to the BMJ BMJ 1996 313 275 283 10.1136/bmj.313.7052.275 8704542 PMC2351717

[B11] Gold MR Cost-effectiveness in health and medicine 1996 New York: Oxford University Press

[B12] Siegel JE Weinstein MC Russell LB Gold MR Recommendations for reporting cost-effectiveness analyses, Panel on Cost-Effectiveness in Health and Medicine JAMA 1996 276 1339 1341 10.1001/jama.1996.03540160061034 8861994

[B13] Nuijten MJ Pronk MH Brorens MJ Hekster YA Lockefeer JH de Smet PA Reporting format for economic evaluation: Part II: Focus on modelling studies Pharmacoeconomics 1998 14 259 268 10.2165/00019053-199814030-00003 10186465

[B14] Vintzileos AM Beazoglou T Design, execution, interpretation, and reporting of economic evaluation studies in obstetrics Am J Obstet Gynecol 2004 191 1070 1076 10.1016/j.ajog.2004.05.021 15507923

[B15] Drummond M Manca A Sculpher M Increasing the generalizability of economic evaluations: recommendations for the design, analysis, and reporting of studies Int J Technol Assess Health Care 2005 21 165 171 15921055

[B16] Ramsey S Willke R Briggs A Brown R Buxton M Chawla A Good research practices for cost-effectiveness analysis alongside clinical trials: the ISPOR RCT-CEA Task Force report Value Health 2005 8 521 533 10.1111/j.1524-4733.2005.00045.x 16176491

[B17] Goetghebeur MM Wagner M Khoury H Levitt RJ Erickson LJ Rindress D Evidence and value: impact on decisionmaking—the EVIDEM framework and potential applications BMC Health Serv Rev 2008 8 270 10.1186/1472-6963-8-270 PMC2673218 19102752

[B18] Davis JC Robertson MC Comans T Scuffham PA Guidelines for conducting and reporting economic evaluation of fall prevention strategies Osteoporos Int 2010 22 2449 2459 21104231 10.1007/s00198-010-1482-0

[B19] Petrou S Gray A Economic evaluation using decision analytical modelling: design, conduct, analysis, and reporting BMJ 2011 342 d1766 10.1136/bmj.d1766 21482590

[B20] Petrou S Gray A Economic evaluation alongside randomised controlled trials: design, conduct, analysis, and reporting BMJ 2011 342 d1548 10.1136/bmj.d1548 21474510 PMC3230107

[B21] Husereau D Drummond M Petrou S Carswell C Moher D Greenberg D Consolidated Health Economic Evaluation Reporting Standards (CHEERS)—Explanation and elaboration: A report of the ISPOR Health Economic Evaluations Publication Guidelines Task Force Value Health 2013 16 231 250 10.1016/j.jval.2013.02.002 23538175

[B22] Moher D Schulz KF Simera I Altman DG Guidance for developers of health research reporting guidelines PLoS Med 2010 7 e1000217 10.1371/journal.pmed.1000217 20169112 PMC2821895

[B23] Moher D Weeks L Ocampo M Describing reporting guidelines for health research: a systematic review J Clin Epi 2011 64 718 42 10.1016/j.jclinepi.2010.09.013 21216130

[B24] Campbell SM Hann M Roland MO Quayle JA Shekelle PG The effect of panel membership and feedback on ratings in a two-round Delphi survey: results of a randomized controlled trial Med Care 1999 37 964 968 10.1097/00005650-199909000-00012 10493474

